# Optimizing base fluid composition for PEMFC cooling: A machine learning approach to balance thermal and rheological performance

**DOI:** 10.1038/s41598-025-11542-5

**Published:** 2025-07-27

**Authors:** Praveen Kumar Kanti, Prashantha Kumar H. G, Nejla Mahjoub Said, V. Vicki Wanatasanappan, Prabhu Paramasivam, Leliso Hobicho Dabelo

**Affiliations:** 1 Institute of Power Engineering, Universiti Tenaga Nasional, IKRAM-UNITEN, Jalan, 43000 Selangor Malaysia; 2 Department of Mechanical Engineering, Rayat Bahra Institute of Engineering and Nano Technology, Hoshiarpur, Punjab India; 3 Department of Aerospace Engineering, Dayananda Sagar University (DSU), Bangalore, 560056 India; 4 Research and Innovation Cell, Bahra University, Distt. Solan, HP, Solan, Waknaghat India; 5 Department of Physics, College of Science, King Khalid University, Abha, 61413 Saudi Arabia; 6 Department of Research and Innovation, Saveetha School of Engineering, SIMATS, Chennai, 602105 Tamil Nadu India; 7 Department of Mechanical Engineering, Mattu University, Mettu-318, Mettu, Ethiopia

**Keywords:** Aluminium oxide, Proton exchange membrane fuel cell, Reduced graphene oxide, Thermal conductivity, Viscosity, Engineering, Mechanical engineering

## Abstract

The Proton Exchange Membrane Fuel Cell (PEMFC) is a highly efficient and eco-friendly technology, making it a pivotal solution for sustainable energy systems. Effective thermal management of PEMFCs is essential, and nanofluids have emerged as superior coolants compared to conventional fluids. Less exploration in PEMFC cooling, particularly using reduced graphene oxide (rGO) suspended hybrid nanofluids, supports the present work on the thermal and rheological properties of rGO-based hybrid nanofluids. The experimental exploration involves five different mixtures of base fluid composition comprising ethylene glycol (EG) and water (W). The hybridization of Al₂O₃ and rGO nanoparticles was performed by dispersing both at four different concentrations in the 50:50 base fluid mixture. The experimental procedure involves evaluation of dispersion stability, viscosity, and thermal conductivity of hybrid nanofluids. The results showed that increasing the EG proportion reduced thermal conductivity while increasing viscosity. The maximum thermal conductivity ratio of 1.23 occurred at 80:20 W: EG for 1 vol% concentration at 60 °C, while the highest viscosity ratio of 1.48 was observed at 20:80 W: EG at 30 °C. The developed correlation for viscosity shows an 11.2% reduction in the coefficient of determination obtained for the thermal conductivity model. This study explores the application of Linear Regression (LR), Decision Tree (DT), and eXtreme Gradient Boosting (XGBoost) models for predicting thermal conductivity and viscosity using experimental datasets. The thermal conductivity model showed that XGBoost has the best predictive power, with Test R² = 0.9941, Test mean square error (MSE) = 0.0000, and Test KGE = 0.9613. XGBoost again beat other models in predicting viscosity, with Test R² = 0.9944, Test MSE = 0.0269, and Test KGE = 0.9903. SHapley Additive exPlanations (SHAP) and Local Interpretable Model-agnostic Explanations (LIME) graphs showed that the model outputs were greatly affected by the base fluid ratio (BFR), temperature, and concentration. This made the model outputs easy to understand both globally and locally. These findings provide valuable insights for designing efficient cooling solutions for PEMFCs, supporting their broader adoption in energy applications.

## Introduction

A PEMFC is an electrochemical device that generates electricity by converting oxygen and hydrogen using a special proton exchange membrane. This device is the key element of clean energy technology due to its high energy conversion efficiency, zero-emission, and fast start up^1^. However, the devices also produce water and a large amount of heat as the by-product. The excessive amount of heat energy produced has a high potential to cause cell degradation and also reduce the life span of PEMFC^2–4^. For instance, Rohendi et al.^3^ mentioned that the degradation of membrane electrode assembly (MEA) at a cell temperature of 80 °C significantly affects the performance characteristic of PEMFC. Therefore, most PEMFC devices require a cooling system utilizing either active or passive cooling or a combination of both mechanisms.

The incorporation of cooling system in PEMFC devices is crucial to maintain a safe temperature condition while maintaining uniform heat distribution. PEMFCs operate efficiently within a range of approximately 60 to 120 °C, balancing performance and durability^4^. Operating below this range slows reactions, reducing efficiency, while exceeding it risks membrane dehydration and thermal degradation, compromising effectiveness and lifespan^5^. Maintaining operation within this band is crucial for optimizing PEMFC performance and lifespan. A recent review by Abubakar et al.^6^ on primary cooling techniques of PEMFC reported that liquid cooling is significantly effective in maintaining the cell temperature compared to air cooling which is less effective due to its dependency on ambient air. However, temperature uniformity remains a major challenge as it affects the MEA^7^. Therefore, researchers are actively working on new generation working fluid to improve the liquid-based cooling strategy for PEFMC, particularly for automotive applications.

Hybrid nanofluid is a new-generation working fluid that offers better thermal characteristics compared to unitary nanofluids or mono nanofluids^8^. A hybrid nanofluid is created by dispersing two distinct types of nanoparticles in a base fluid, typically consisting of commonly used working fluids like water, EG, or their combinations at different ratios. These base fluids have wide-ranging applications across various industries^9–11^. Hybridization represents an innovative strategy aimed at merging the properties of two different materials to produce a unified entity with enhanced and unique characteristics. The principle behind hybrid nanofluids involves altering the base fluid’s properties by integrating two different nanofluids, each possessing diverse material properties. However, it’s crucial to acknowledge that assessing the suitability of base fluid combination in enhancing the thermophysical properties of hybrid nanofluids because most literature work is focused on optimizing the nanoparticle ratios and concentration in base fluid to achieve enhancement in thermal conductivity^12^.

The composition of base fluid plays an important role especially when the mixture of base fluid consists of conventional cooling fluid such as water and EG. The composition of water and EG has a high potential to alter the thermophysical properties of hybrid nanofluid. Labib et al.^13^ conducted a numerical investigation on the effect of the base fluid and hybrid nanofluid consisting of Al_2_O_3_-CNT nanoparticles in the forced convection heat transfer application. They found that EG performed much better compared to water as the base fluid and the Al_2_O_3_-CNT combination resulted in significant heat transfer enhancement. Yet, for the mixture of base fluid containing water and EG, the most common ratio investigated is 60:40 and this composition resulted in significant improvement in thermal conductivity for nanoparticle combination of Al_2_O_3_-CuO^10,14^, Al_2_O3-Fe_2_O_3_^15^, Al_2_O_3_-TiO_2_^16^. However, Selvarajoo et al.^17^ found that the mix ratio of 60:40 produces unstable hybrid nanofluid for GO-Al_2_O_3_ combination and the base fluid ratio of 80:20 (Water: EG) was able to produce stable hybrid nanofluid with 4.3% enhancement in thermal conductivity. Meanwhile for Al_2_O3-SiO_2_-TiO_2_ composition, the hybrid nanofluid with a base fluid ratio of 70:30 produced about 15% enhancement in radiator cooling capacity.

Based on past research findings, it is evident that the composition of base fluid varies according to the types of nanoparticles and their composition. Even though most base fluids and their ratios are suitable for Al_2_O_3_ nanoparticles, the hybridization of Al_2_O_3_ with 2 dimenisonl material such as rGO requires optimum base fluid composition to provide optimum thermal performance and stability. rGO belongs to the class of “graphene materials” and is produced through numerous methods, like as chemical, thermal, and UV or IR irradiation, all aimed at reducing the oxygen content^18,19^. With its notable attributes of high conductivity and stability, rGO emerges as a promising option for applications in energy storage and biomedical fields^20,21^, owing to its greater surface-to-volume ratio, higher electron mobility, and electrochemical potential^22–24^.

Singh et al.^25^ and Vardaru et al.^26^ used rGO for the hybrid nanofluid preparation and reported that the addition of rGO enhances the thermal conductivity of the hybrid nanofluid. Dinesh et al.^27^ and Islam et al.^28^ highlighted in their review paper recent advances in using nanofluids for enhanced thermal management in PEMFCs, emphasizing their role in improving heat transfer, reducing radiator size, and maintaining long-term stability. It also identifies gaps in understanding electrical properties and calls for standardized methods and deeper exploration of smart nanofluids for scalable, efficient cooling solutions.

Genc et al.^29^ explore Fe₃O₄-water nanofluids for PEMFCs cooling, emphasizing the need for high thermal performance, stability, and low electrical conductivity. Optimal results were achieved at 0.4% mass ratio, with up to 19% heat transfer enhancement. Zakaria et al.^30^ used Al₂O₃ nanofluids in water–EG blends (0–100%) for PEMFC cooling and they reported that at 0.5%, thermal conductivity dropped from 0.6478 (0% EG) to 0.2816 W/m-K (100% EG). Solatnai et al.^31^ numerically analyzed four Tesla valve cooling designs in PEMFCs using different hybrid nanofluids. The reverse Tesla valve improved heat transfer by 15% and achieved better thermal uniformity. Ag–MgO nanofluid showed the best performance with a 4% lower temperature difference across the cooling plate.

Even though, Al_2_O_3_ has been extensively studied for its use in hybrid nanofluids, mainly because it is cost-effective, readily available, and possesses anti-corrosive properties^24,32–37^. Nevertheless, combining Al_2_O_3_ with graphene derivatives offers the possibility of further enhancing properties, which deserves careful investigation. Furthermore, the lack of comprehensive experimental research focusing on the formulation of base fluid ratios with NP concentration motivates the present research work. Also, the absence in aspects of PEMFCs for different volume proportions of base fluids investigation employing rGO and Al_2_O_3_ combination provides a strong base support for the experimental work. Achieving the right balance of hybrid nanofluid properties by identifying the optimum base fluid composition is crucial for realizing their potential benefits as cooling agents in PEMFCs.

Modeling physicochemical properties such as thermal conductivity and viscosity based on experimental parameters from PEMFC presents inherent complexity due to the nonlinear interactions among variables like BFR, concentration, and temperature. These traits are typically affected by multidimensional dependencies and hidden patterns that are hard to see using standard linear modelling methods. Also, getting high-quality experimental data takes a lot of time, accuracy, and money, therefore it’s important to develop prediction models that can work effectively in a wide range of situations. Machine learning (ML) methods like DTs, LR, and eXtreme Gradient Boosting provide strong frameworks for effectively capturing these complicated connections. ML models can look at huge amounts of data, find hidden patterns, and make accurate predictions without having to use equations^15^. But one big problem with conventional ML models, particularly high-performing ensemble models like XGBoost, is that they are black boxes, which makes it hard to figure out how they get to their decisions. This lack of openness may make it hard for people to trust and use the technology more widely, especially in science and engineering where being able to explain things is quite important. To fix this, explainable ML techniques like SHAP and LIME have become quite important. SHAP helps us understand how each input variable affects the model’s predictions for all samples, whereas LIME helps us understand how the model behaves around a single instance by simplifying it. These approaches work together to close the gap between prediction accuracy and interpretability^32^. This lets researchers not only anticipate outputs but also explain how each parameter affects them. This makes ML-driven modelling of complicated thermofluidic systems more scientifically reliable.

The present work focused on the preparation and evaluation of thermophysical properties of a novel rGO-Al_2_O_3_ (50:50) hybrid nanofluid with varying concentrations ranging from 0 to 1 vol%. Hybrid nanofluids were prepared using water and EG blends at five different blend ratios (20:80, 40:60, 50:50, 60:40, and 80:20) and experimented for their thermal conductivity, viscosity and dispersion stability. The thermal and rheological investigation is performed at a temperature range of 25 to 60^o^C. Additionally, morphological and structural characterization of nanoparticles is performed using X-ray diffraction (XRD) technology and transmission electron microscopy (TEM) techniques. Regression analysis was used to develop a new correlation for predicting viscosity and thermal conductivity of the hybrid nanofluids, enabling better insight into their behavior, improved dispersion stability, and enhanced thermophysical properties for PEMFC use. The goal of the research is to test and compare how well regression, DT, and XGBoost models work in these settings. It also focuses on utilizing SHAP and LIME to explain model choices to make sure they are transparent and robust in prediction.

## Methodology

### Materials

The experimental materials comprised Al₂O₃ and rGO nanoparticles procured from Sigma Aldrich, USA, with average particle sizes of 13 nm and 5–50 nm, respectively, as per supplier specifications. Water and ethylene glycol were sourced from RS Components Sdn. Bhd., and Cetyltrimethylammonium bromide (CTAB) was used as a surfactant to ensure nanofluid stability.

The reason for the selection of CTAB (cationic surfactant) was driven by strong results on dispersion stability as reported in various literature for rGo and Al_2_O_3_ nanofluids. CTAB can enhance the dispersion of rGO and Al_2_O_3_ nanoparticles in base fluid due to its long hydrophobic tail and positively charged head group which can adsorb on rGO surface and prevent restacking of rGO. This ensures the homogeneity of rGO in base fluid mixtures. Besides, CTAB acts as a compatibilizer between hydrophobic rGO and hydrophilic Al₂O₃, enabling uniform suspensions of rGO-Al_2_O_3_ in base fluid. Besides, Bhavin et al.^38^ reported excellent stability and thermal property enhancement in their research work employing CTAB as a surfactant for aqua Al_2_O_3_ nanofluids. Table 1 provides a detailed summary of the material properties used in the experimental study.

**Table 1:** Comprehensive specifications and properties of the materials used.

Properties	Nanoparticles		Surfactant
	Al_2_O_3_	rGO	CTAB
Purity (%)	99.8	99.8	96
Particle size (nm)	13	5–50	-
Density (kg/m^3^)	3950	1850	2300
Specific heat (J/kg K)	775	710	-
Thermal conductivity (W/m K)	36	2600	-

### Nanofluid Preparation

The hybrid nanofluid synthesis followed a two-step method. First, water and EG were mixed in mass ratios of 80:20, 60:40, 50:50, 40:60, and 20:80 and stirred magnetically for 90 min. A 50:50 nanoparticle blend was then weighed using a digital balance (AG204 Mettler Toledo) and added to the solution to obtain hybrid nanofluid concentrations between 0.25 and 1 vol%^16^. CTAB was added in a 1:10 ratio to the NPs, and the mixture was ultrasonicated using a 20 kHz probe at 55% amplitude for 1.5 h, with 15-second pulses and 5-second rests. The process was conducted in an ice bath to maintain 30–35 °C and avoid overheating. A thermometer was used to monitor temperature. Finally, the solution was ultrasonicated in a bath at 40 kHz for 3 h.

### Characterization

The crystal structure, grain size, and composition of the nanoparticles were examined using XRD with a Shimadzu XRD-6000 diffractometer. Both scanning electron microscopy (SEM) and TEM imaging, performed with TESCAN VEGA and JEOL equipment, respectively provided detailed insights into nanoparticle morphology and dispersion. Zeta potential of the hybrid nanofluid was measured using a Brookhaven Zeta Plus analyzer to assess dispersion stability, with five replicate readings taken. pH values of water and hybrid nanofluid were measured at 25–26 °C using a Hanna HI98103 pH meter, calibrated with a pH 7 buffer before each use. Only stable readings were recorded to ensure accuracy.

### Thermophysical property measurement

The viscosity of hybrid nanofluids, which influences friction factor and pressure drop in PEMFCs, was measured using an Anton Paar Rheometer. Around 16 ml of hybrid nanofluid was placed in the cylinder jacket, where a rotating spindle gauged fluid resistance. A thermal water bath was used to control temperature between 25 and 60 °C during testing.

Incorporating nanofluids into PEMFC cooling systems or electrolytes significantly improves heat management, temperature uniformity, and overall system efficiency. Thermal conductivity of produced fluids was measured using a Tempos thermal analyzer with a 0.06 m KS-1 sensor, based on the transient hotwire method and a 5% uncertainty margin. The sensor emitted controlled heat to minimize convection errors, and calibration was done using glycerine. Measurements were performed across a temperature range of 25–60 °C.

### Machine learning for model prediction

#### Linear regression

One of the simplest forms of supervised machine learning is LR. It operates on the principle that there is a linear connection between the input data and the target variable. It is a kind of parametric model, which means it uses a set of coefficients to identify the best-fit line or plane in multidimensional space. The algorithm works by employing a technique called ordinary least squares to minimize the residual sum of squares between the observed and anticipated values. LR is easy to understand since it is simple, but it also means it can’t describe complicated or nonlinear data patterns^15^. There aren’t many hyperparameters in LR, but you may use regularization methods like L1 (Lasso) or L2 (Ridge) to limit overfitting by penalizing big coefficient values. These penalties make the model less complicated, which helps it work better on data it hasn’t seen before. The science behind it is figuring out the appropriate coefficients for each characteristic that will make the error term as little as feasible. Linear regression has certain flaws, but it may still be a useful technique for datasets with distributions that are easy to work with and not too much noise. It is a good standard for most machine learning jobs and helps you quickly figure out whether a more complicated model is even needed^23,32^.

#### Decision tree

Decision tree (DT) is a type of supervised learning technique that doesn’t need any parameters and works well for both classification and regression problems. The model is set up as a sequence of hierarchical choices. Each internal node represents a condition or feature split, and each leaf node gives a result or prediction. During training, the dataset is broken up again and again depending on feature thresholds that do the greatest job of lowering impurity. This is commonly done using measures like the Gini index or mean squared error. Because they look like how people make decisions, decision trees are easy to understand. They are very strong because they can represent connections that aren’t straight lines and don’t change as the data is scaled. But they are likely to overfit, particularly if the tree becomes too deep or too many nodes are added. That’s why hyperparameters like max depth, minimum samples per leaf, and splitting criteria are so important for keeping the tree simple and making it better at generalizing^23,32^. The main concept is to split the data up in a manner that minimizes the variation within each group, which makes predictions more accurate. Decision trees are more flexible than linear models since they don’t imply that the function that maps input to output has a certain shape. They may also be sensitive to slight changes in data, which can modify the structure a lot. Tree-based ensembles like bagging and boosting are employed to get around it. Still, a well-tuned decision tree is useful since it is easy to understand and works quickly on medium-sized datasets. It also helps you understand which factors have the most effect on the forecasts by showing the importance of each attribute^15,23^.

#### Extreme gradient boosting

XGBoost is a supervised ML method that uses the notion of boosting to learn from a group of examples. It builds a sequence of decision trees one after the other, with each new tree trying to fix the mistakes produced by the trees that came before it. Boosting combines each tree into a single model that builds on what has previously been learnt, whereas bagging approaches create trees separately. Instead of one big tree, the structure has many little trees. The output is a weighted sum of each tree’s forecast. Gradient descent optimization is the theory underlying XGBoost. It employs the gradients of the loss function to figure out how to change the model at each step. There are a number of hyperparameters that have a big effect on how well XGBoost works. The learning rate regulates how much each tree’s correction changes the final output, while the max depth limits how complicated each tree may become. Subsample and colsample by tree are examples of parameters that control randomization, which helps keep the model from overfitting. Regularization parameters like alpha and lambda punish trees that are too complicated, making the model more useful in general. XGBoost is noted for being fast and efficient since it builds trees in parallel and can manage missing data on its own. The model handles bias and variation well since it is both iterative and additive. It works well in a lot of different situations, even when there is noise or outliers since it is founded on science and uses boosting and gradient-based optimization^23,45^.

### Explainable machine learning

#### Shapley additive explanations

SHAP, or Shapley Additive Explanations, is a model-agnostic explainable machine learning technique that helps in understanding how each feature contributes to a model’s prediction. It shows how each attribute affects a model’s prediction. The aim behind Shapley’s values in cooperative game theory is to equitably divide the “payout” (in this example, the model’s prediction) among the players who contributed (the input characteristics). SHAP gives each characteristic a significance value for a certain prediction by figuring out how much that feature adds to the forecast on average over all feasible combinations. SHAP breaks down a forecast into parts that add up to the output, which makes the interpretation consistent and accurate in a small area. To get SHAP values, you start with a reference or baseline prediction and then see how much adding a certain feature alters the outcome. Computing accurate SHAP values may be quite costly, particularly for big models. That’s why approximations are employed in practice [46]. SHAP is used after the model is developed, therefore it doesn’t change how the model is learned. The main reason SHAP is useful in research like this is that it connects feature values directly to prediction behavior. This lets users not only see what the model predicts but also why it does so. The original model’s hyperparameters might indirectly alter SHAP by modifying the learned structure, which then changes how features are attributed. SHAP is one of the most theoretically sound interpretability tools since it is based on fair allocation principles and additive feature attributions. It may provide both global insights, like feature rating, and local insights, like explanations for individual samples. This makes it useful in situations where trust and understanding are vital ^45,46^.

#### Local interpretable Model-agnostic explanations

LIME, or Local Interpretable Model-agnostic Explanations, is another popular method used to interpret the predictions of machine learning models. The main concept behind LIME is to use a simpler, more understandable model, such as a linear model, to get close to a more complicated model in a narrow area around a given prediction. LIME doesn’t explain the whole model globally; instead, it makes local explanations that show how the model acts around a certain data point. To achieve this, LIME makes a small change to the input sample to create a neighborhood of similar data points, and then it looks at the model’s predictions for each of these changes. Then it fits a basic model to this area to get a rough idea of the complicated decision boundary^15,23^. LIME’s structure includes sampling, weighting, and fitting. Proximity-based weighting makes sure that points that are closer to the original sample have more of an impact on the explanation. This means that the approach may be used with any model and is versatile since it doesn’t need access to internal model structures or gradients. The number of samples produced, the kernel width for distance weighting, and the kind of surrogate model are all hyperparameters that affect how accurate and easy to understand LIME’s output is. LIME is based on local approximation and weighted regression, which lets you look at the model as a black box while yet understanding how each choice was made. However, LIME explanations might change from run to run because of unpredictability in sampling. This can make them less reliable if they aren’t properly calibrated^32^. In fields like material science, LIME may be used to figure out which properties are most important for a certain prediction. This can assist confirm or challenge how a model works. It is particularly helpful when you need to explain choices to people who aren’t experts or when you need to check that the model’s behaviour matches what is expected in the domain ^46^.

## Results and discussion

### Characterization

**Fig. 1:**
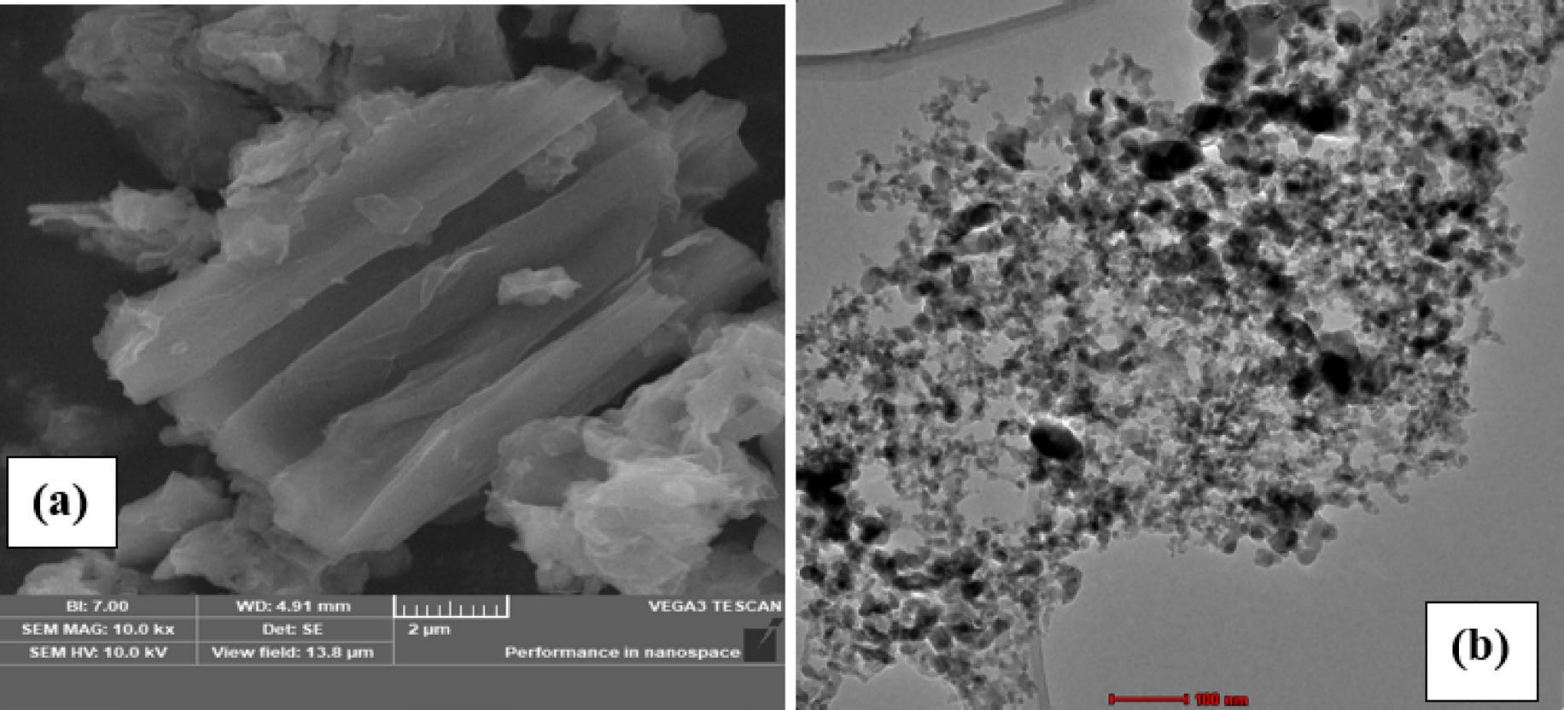
Morphological characterization of nanoparticles: *(***a***)* SEM image of rGO and *(***b***)* TEM image of Al₂O₃ nanoparticles.

Figure 1(a) shows the SEM image of rGO, highlighting its thin, layered, and flake-like morphology with lateral dimensions around 4–5 nm, indicating a high surface area favorable for thermal transport. Figure 1(b) displays TEM image of Al₂O₃ nanoparticles, confirming their uniform spherical shape and good dispersion. The average particle size of Al₂O₃ is approximately 11 nm, as calculated from TEM analysis, which closely agrees with crystallite size obtained from XRD, suggesting high purity and crystallinity of the sample. These morphological features are critical for improving the stability and thermal performance of the resulting hybrid nanofluid.

**Fig. 2:**
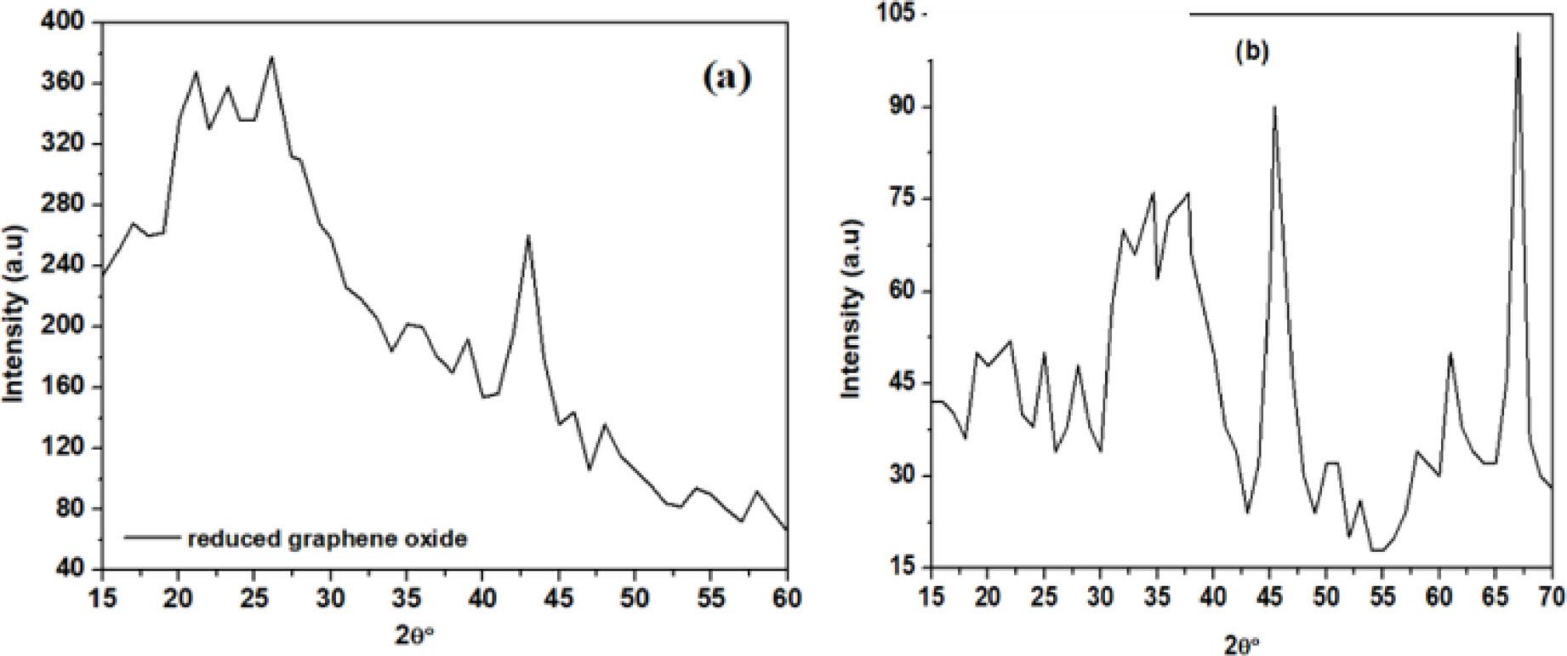
XRD profile of (**a**) rGO (**b**) Al_2_O_3_ recorded for 2θ range of 15–70°.

**Fig. 3:**
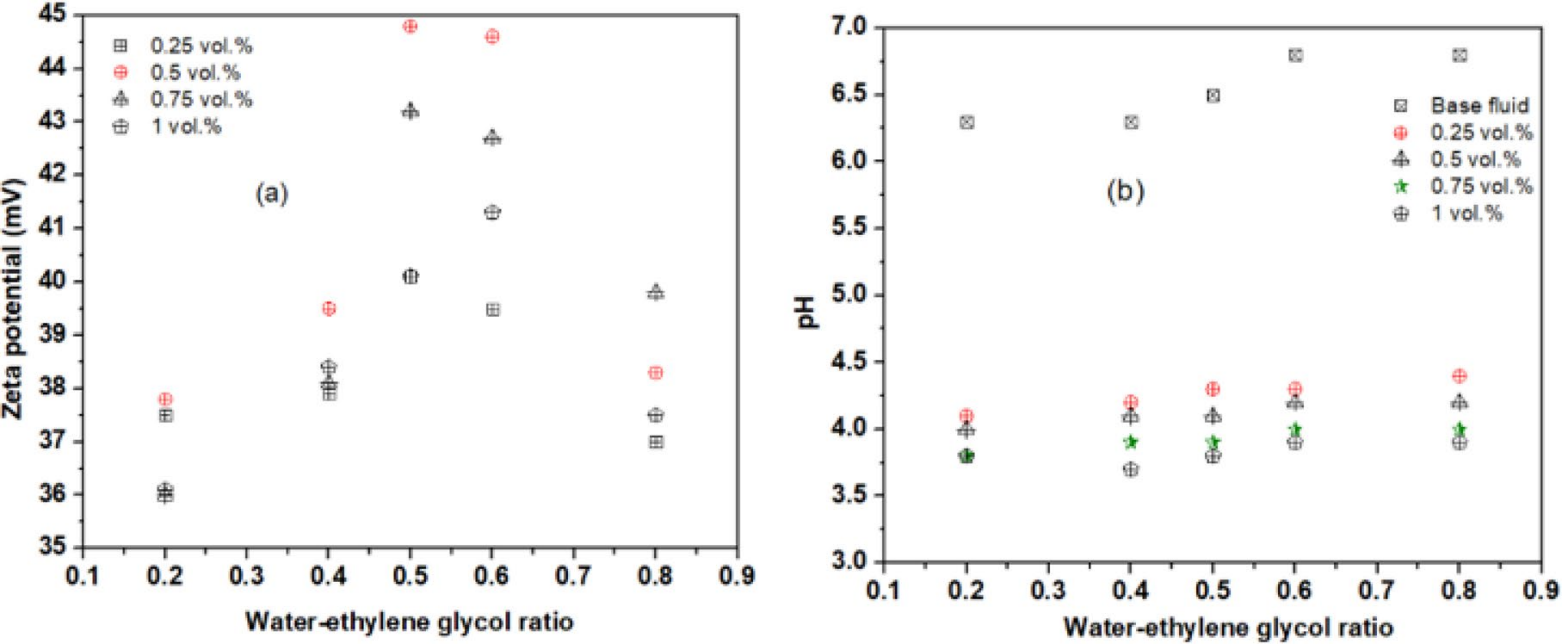
(**a**) Variation of Zeta potential (mV) and (**b**) pH value of rGO-Al_2_O_3_ hybrid nanofluid.

Figures 2(a) and (b) illustrate the XRD patterns of rGO and Al₂O₃ nanoparticles, respectively, obtained using Cu-Kα radiation over a 2θ range of 15°–70° with a step size of 0.02°. In Fig. 3(a), rGO exhibits a weak, broad peak between 2θ = 20°–30°, along with a minor peak near 43°. The appearance of a distinct peak at 2θ = 26° confirms the reduction of GO to rGO, indicating the presence of small crystallites and few-layered structures with residual oxygen-containing groups. Figure 2(b) shows four major diffraction peaks for Al₂O₃ at 2θ values of 34.64°, 37.78°, 45.37°, and 67°, consistent with the crystalline phase identified by JCPDS card no. 46-1215^15^. The broadness of these peaks suggests the presence of fine crystallite sizes, with an average size estimated to be below 11 nm using the Debye–Scherrer equation.

### Stability and pH of rGO-Al_2_O_3_ hybrid nanofluids

The stability of rGO–Al₂O₃ hybrid nanofluid at varying base fluid ratios and concentrations was assessed using a Brookhaven Zeta Plus analyzer. Figure 3 (a) presents the zeta potential values, which indicate the colloidal stability of the suspension. According to literature^22,23,33,34^, hybrid nanofluids with zeta potential magnitudes ≥ 40 mV is considered highly stable. All samples showed values above 36 mV, with visual confirmation of stability. The 60:40 and 50:50 base fluid ratios exhibited the highest stability, recording peak zeta potentials of − 44.8 mV and − 44.6 mV, respectively. The strong negative charge promoted interparticle repulsion, minimizing agglomeration and enhancing suspension stability. Concentration had minimal effect on zeta potential; however, the 0.5% concentration consistently showed the highest stability across all fluid ratios. These findings align with results reported by Khesarubini et al.^17^, who observed a maximum zeta potential of − 49.7 mV for Al₂O₃–GO hybrid nanofluids at 1% concentration. Stability is critical for ensuring the reliability of nanofluids in practical heat transfer applications.

The pH of rGO–Al₂O₃ hybrid nanofluid plays a critical role in determining dispersion stability. As shown in Fig. 3(b), the base fluid mixtures (water/EG) initially exhibit pH values between 6.3 and 6.8. Upon the addition of rGO–Al₂O₃ NPs, the pH drops significantly into the acidic range. For a 50:50 base fluid ratio, a 0.25% NP concentration caused a pH reduction of approximately 36.7%. The largest drop was observed at an 80:20 (EG/water) ratio with 1% concentration. This decrease is primarily due to rGO, which has a lower pH (∼3.3) compared to Al₂O₃ (∼6.0) as reported by Khesarubini et al.^17^. The measured pH range of 3.7–4.4 lies between the isoelectric points (IEP) of rGO (~ 2.5)^21^ and Al₂O₃ (~ 7.1)^34^, promoting strong electrostatic repulsion between particles and enhancing stability. This underscores the importance of adjusting pH away from the IEP to maintain stable nanofluid suspensions.

### Validation

**Fig. 4:**
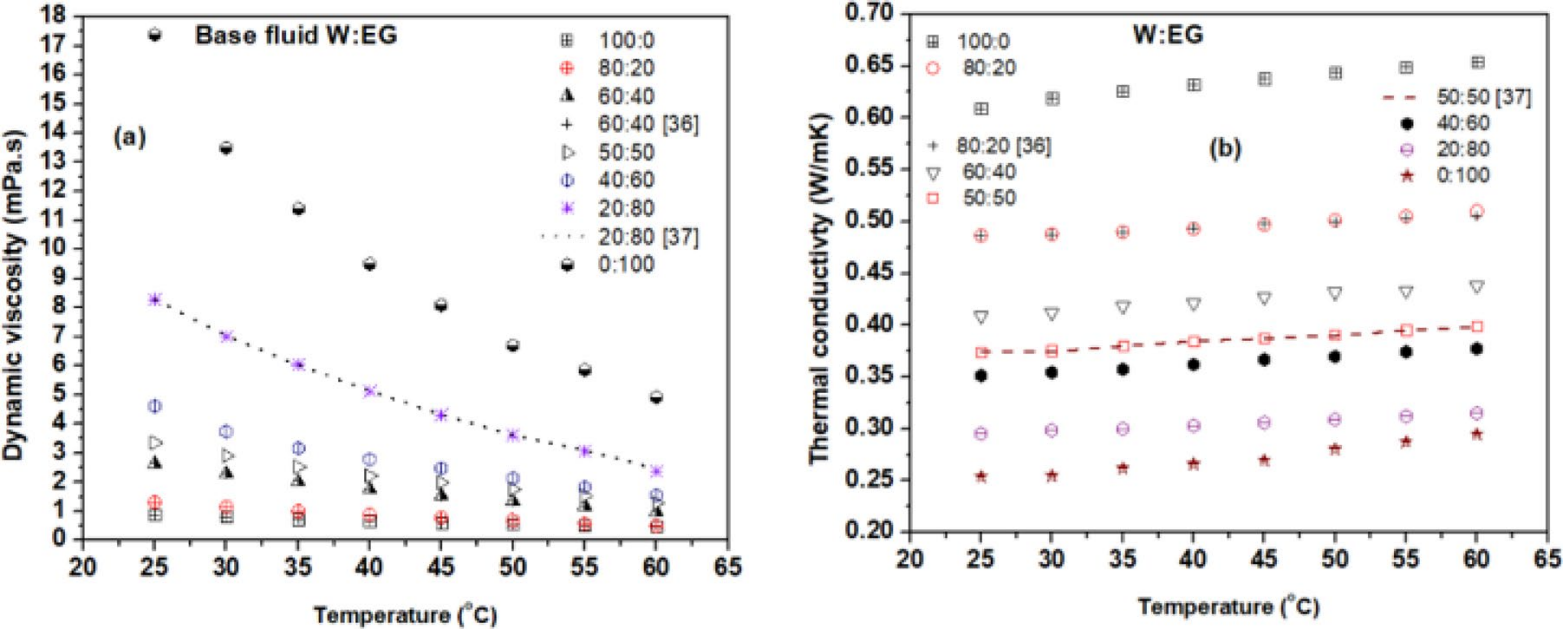
Comparison of experimental base fluid data with literature (**a**) dynamic viscosity and (**b**) thermal conductivity.

Initial validation of experimental data is essential to ensure instrument accuracy and reliable measurements^35^. This was achieved by comparing the measured viscosity and thermal conductivity values of W: EG mixtures with established literature. Figure 4(a) shows experimental viscosity data across various ratios, validated against Sawicka et al.^36^ for 60:40 and ASHRAE^37^ for 20:80 mixtures. Figure 4(b) presents thermal conductivity measurements for different ratios, with 80:20 and 50:50 mixtures compared to the same references over 25–60 °C. The minimal deviations observed confirm the accuracy of the experimental setup.

### Viscosity

**Fig. 5:**
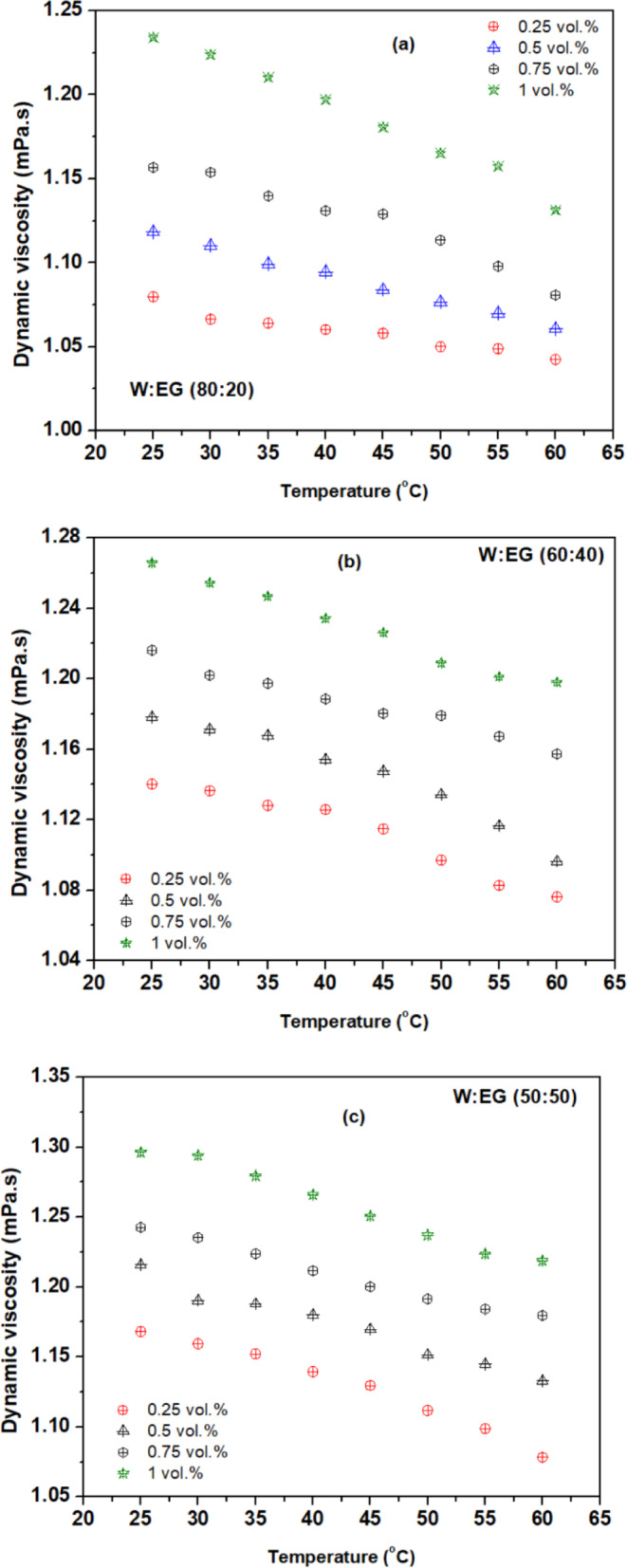

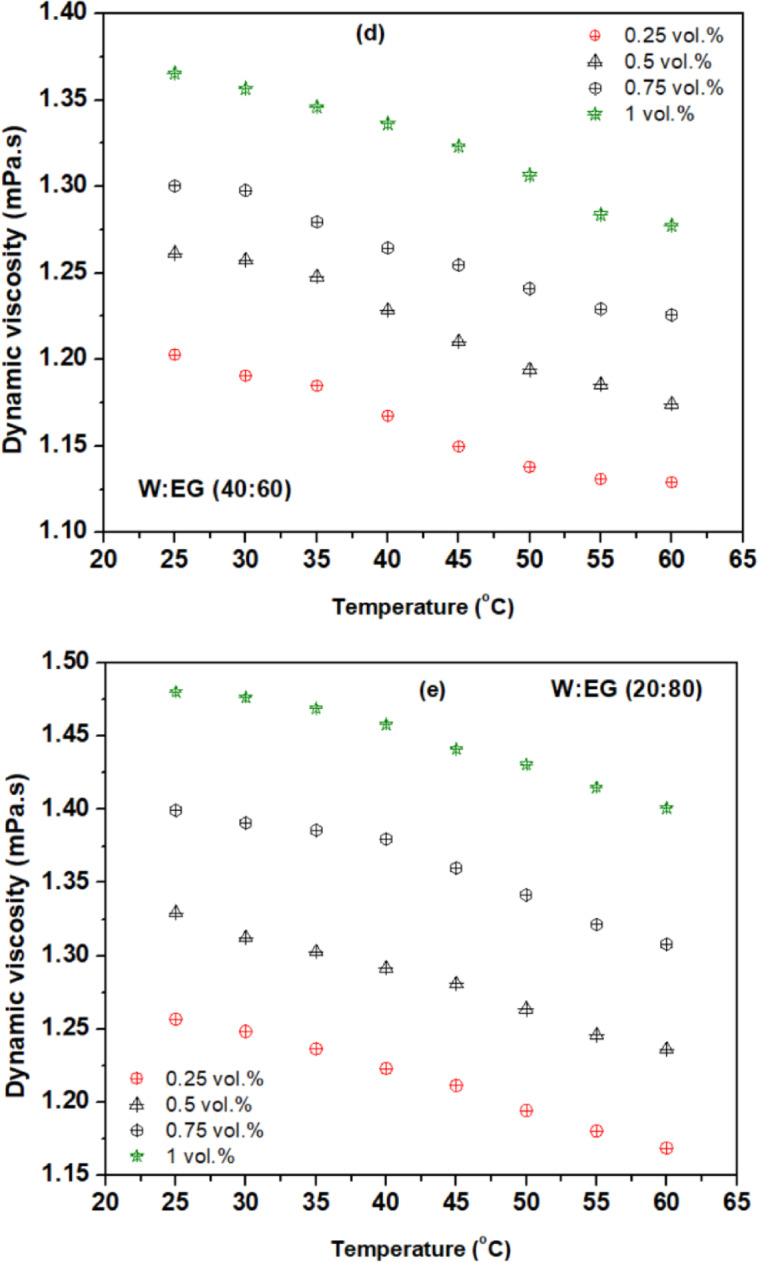
Viscosity ratio of hybrid nanofluid with temperature for base fluid ratios of (**a**) 80:20 (**b**) 60:40 (**c**) 50:50 (**d**) 40:60 (**e**) 20:80.

Figures 5(a) to (e) depict the viscosity ratio ($\:\frac{{\mu\:}_{nf}}{{\mu\:}_{bf}}$) variations at different concentrations and temperatures for various blend ratios of base fluids. The observed trend reveals that the viscosity of the nanofluids surpasses that of the water and escalates with growing concentration. This phenomenon is attributed to the heightened internal shear stress within the fluid upon dispersion of nanoparticles into the base fluid^35^. Notably, the escalation in viscosity concerning particle concentration notably exceeds that of the water, particularly evident at higher volume concentrations^39^. The observed decrease in the viscosity of nanofluids with increasing temperature can be attributed to the corresponding rise in internal energy within the fluid molecules and nanoparticles. This elevated temperature leads to heightened molecular motion, overpowering intermolecular binding forces. Consequently, the intermolecular and interparticle spacing widens, while attractive forces diminish. Moreover, the viscosity of the fluid is contingent upon the internal friction between molecules, dictated by intermolecular attraction. As temperature increases, the distance between liquid molecules and nanoparticles expands, gradually weakening intermolecular attraction and reducing internal friction. Ultimately, this interplay results in a decrease in fluid viscosity^39,40^. The maximum viscosity ratio of 1.26, 1.33, 1.40 and 1.48 is observed for a concentrations 0.25, 0.5, 0.75, and 1 vol% respectively, at 25^o^C compared to the base fluid (W: EG) mixture ratio of 20:80. Similarly, the minimum viscosity ratio of 1.042, 1.06, 1.08 and 1.13 is observed for a concentrations 0.25, 0.5, 0.75, and 1 vol% respectively, at 60^o^C compared to the base fluid (W: EG) mixture ratio of 80:20.

The viscosity analysis extends to encompass various mixture ratios of W: EG-based nanofluids. The rise in viscosity is contingent upon the proportion of W and EG within the base fluids under study. Notably, nanofluids formulated with a higher proportion of EG in the base fluid mixture ratio exhibit more pronounced viscosity enhancement compared to those based on water. For instance, with base fluid blend ratios of 20:80 (W: EG), suspension of 1% volume concentration of rGO-Al_2_O_3_ nanoparticles resulted in a 48% rise in viscosity for ambient temperature conditions of 25 °C. Consequently, when a similar composition of nanoparticles is dispersed in a base fluid blend ratio of 80:20 (W: EG), the rise in base fluid viscosity is only 13% at a temperature condition of 25 °C. The significant increase in viscosity has a direct impact on the pumping power and pressure drop, impacting the overall system efficiency and performance of the PEMFC cooling system. According to Bargal et al.^41^, the net electrical output of the PEMFC system reduced significantly as the pumping power increased mainly due to the excess energy drawn from the system to maintain the fluid flow.

Despite variations in the W: EG blend ratio, all nanofluids showcased viscosity amplification with increasing concentrations, yet this effect diminished with rising temperatures. A 35 °C rise in temperature of hybrid nanofluid with 1% concentration and base fluid blend ratio of 20:80 (W: EG) resulted in about 71.7% decrease in viscosity. Since the PEMFC cooling system often operates at a temperature of 60–80 °C, the heat energy able to reduce the flow resistance and diminish the pressure drop and rise in pumping power. Therefore, the application of rGO-Al_2_O_3_ hybrid nanofluid with blend ratio of 20:80 has a high potential to improve the efficiency of PEFMC system and used in transportation sectors that utilizes high power PEMFC stacks (> 10 kW) [48].

### Thermal conductivity

**Fig. 6:**
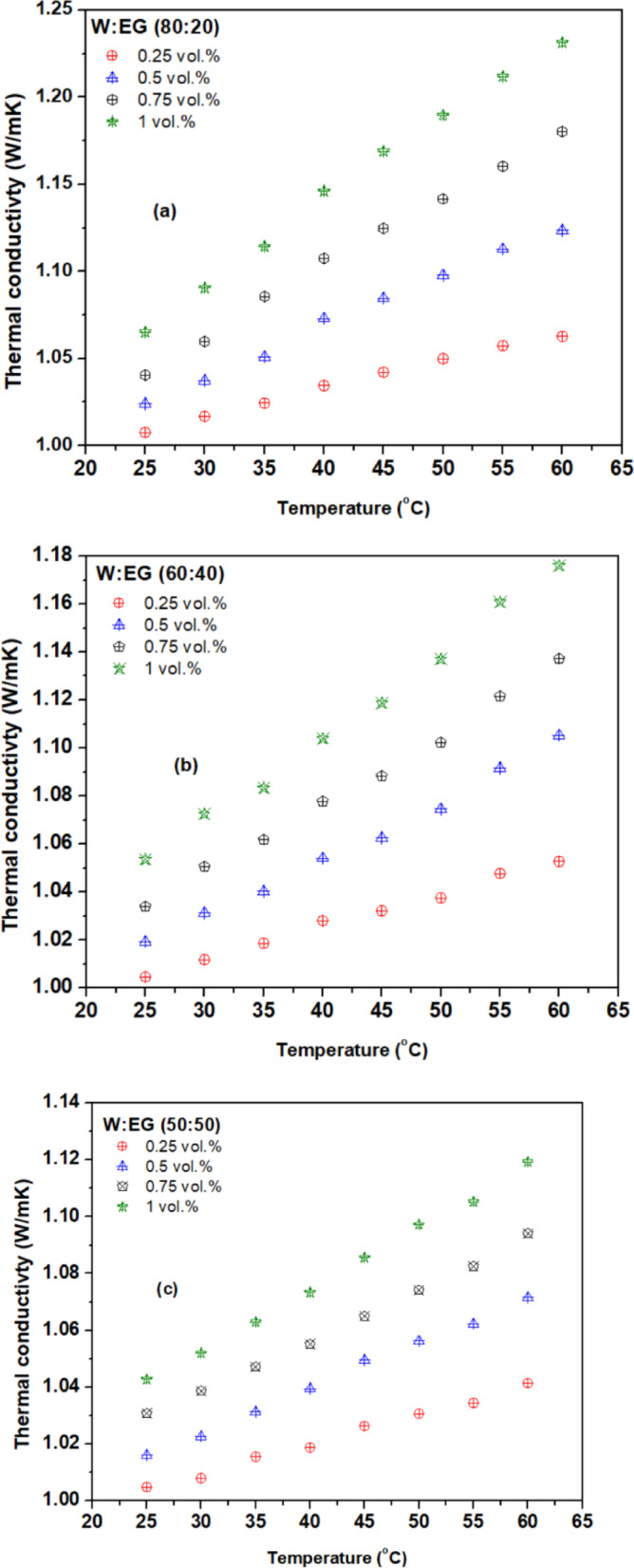

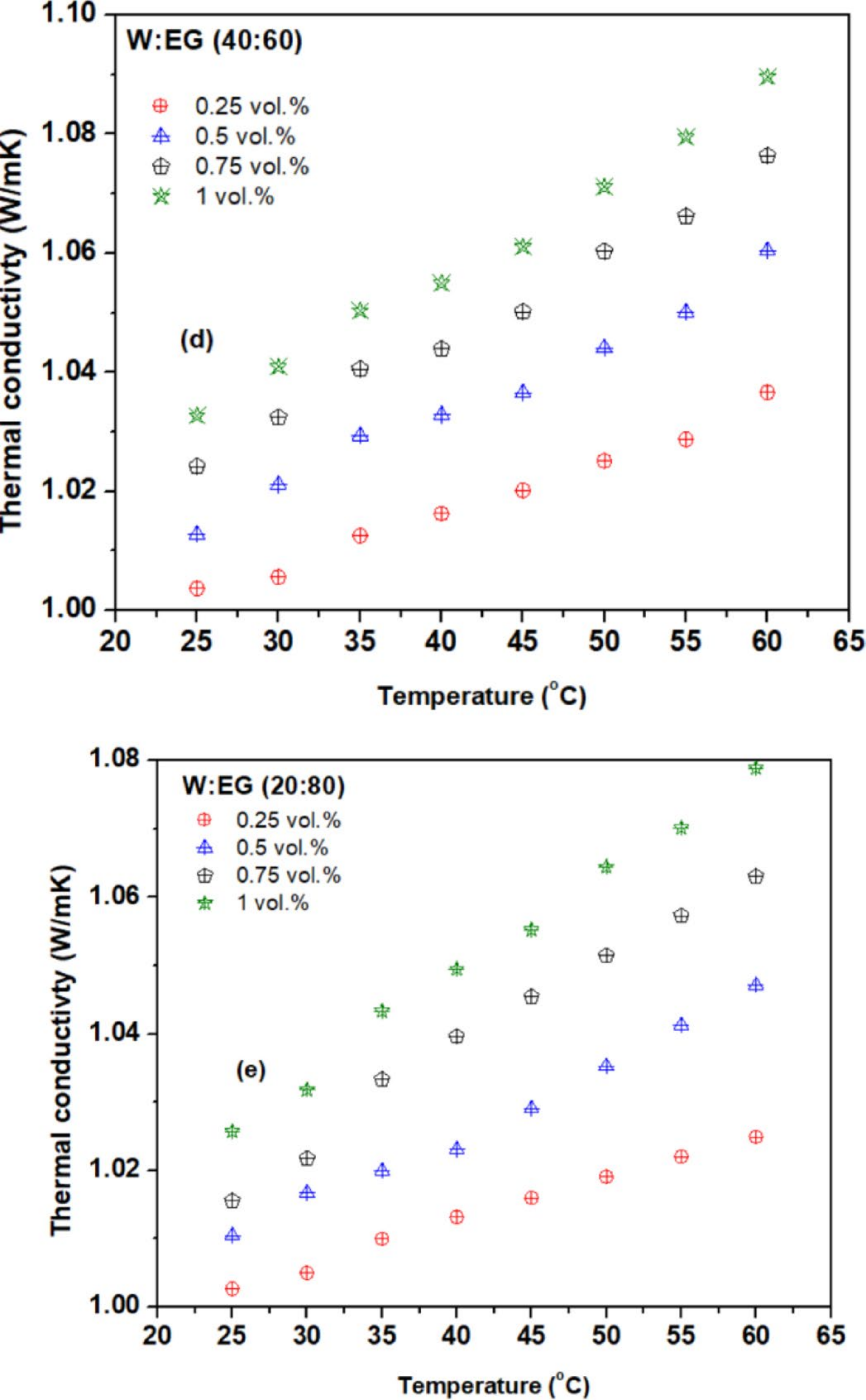
Thermal conductivity variation with temperature for base fluid blend ratios of (**a**) 80:20, (**b**) 60:40, (**c**) 50:50, (**d**) 40:60, and (**e**) 20:80.

Figures 6 (a) to (e) illustrate the variation of thermal conductivity ratio ($\:\frac{{k}_{nf}}{{k}_{bf}}$) of hybrid nanofluid across different concentrations and temperatures using various base fluid mixture ratios, revealing improvements with increasing temperature and concentration compared to their respective base fluid mixtures. This enhancement can be attributed to possible mechanisms like nanoparticle migration, aggregation, base fluid properties, thermophoresis, molecular level liquid stacking at the nanoparticle-liquid interface, and micro-convection driven by Brownian motion with the addition of a greater number of nanoparticles in the base fluid^42,43^. Additionally, as the temperature rises, thermal conductivity increases due to decreased viscosity and intensified Brownian motion of nanoparticles, underscoring the multifaceted influence of particle concentration, temperature, and thermal conductivity of base fluid on enhancement of nanofluid thermal conductivity. The highest thermal conductivity ratio of 1.063, 1.123, 1.18, and 1.23 is observed for a concentrations 0.25, 0.5, 0.75, and 1 vol% respectively, at 60^o^C compared to the base fluid (W: EG) mixture ratio of 80:20. Similarly, the thermal conductivity ratio of 1.01, 1.011, 1.016, and 1.026 is observed for a concentrations 0.25, 0.5, 0.75, and 1 vol% respectively, at 25^o^C compared to the base fluid (W: EG) mixture ratio of 20:80.

Thermal conductivity enhancement varies notably between water and EG base fluids, underscoring the importance of base fluid selection. Water-based hybrid nanofluids generally exhibit higher thermal conductivity improvements compared to EG or W: EG mixtures. While all hybrid nanofluids showed increased thermal conductivity with rising temperature and concentration, the W: EG (80:20) mixture achieved the highest thermal conductivity, whereas 20:80 recorded the lowest. This highlights the direct influence of base fluid thermal conductivity on hybrid nanofluids performance. Water’s superior thermal conductivity is attributed to its polar molecular structure and strong hydrogen bonding, which facilitates efficient thermal energy transfer. In contrast, EG has weaker hydrogen bonding and lower polarity, limiting its thermal conductivity despite its higher density. Therefore, molecular interactions play a more critical role than density in determining thermal conductivity.

### Regression equations

Regression analysis was performed on the experimental thermal conductivity and viscosity data using Microsoft Excel to derive predictive models for rGO–Al₂O₃ hybrid nanofluids across different base fluid ratios. The independent variables considered were base fluid ratio (R), temperature (T °C), and nanoparticle concentration (vol%), while thermal conductivity and viscosity served as dependent variables. A regression model was employed for both viscosity (R^2^ = 0.93) and thermal conductivity (R^2^ = 0.97) prediction, the resulting equations are provided below.

For viscosity,

1 $$\begin{aligned} &\:{{\upmu\:}}_{\:predicted\:}=18.42-20.45{\it R}-0.351T+4.22{\it V}+0.001346{\it T}^{2}\\ &\quad\quad\quad\qquad-0.60217{\it V}^{2}+0.3123{\it RT}-3.166{\it RV}-0.0289{\it TV}\end{aligned}$$

For thermal conductivity,

2 $$k_{predicted}= 0.1515+0.3672{\it R}+0.001422{\it T}+0.0386{\it V}$$

### Performance enhancement ratio (PER)

**Fig. 7:**
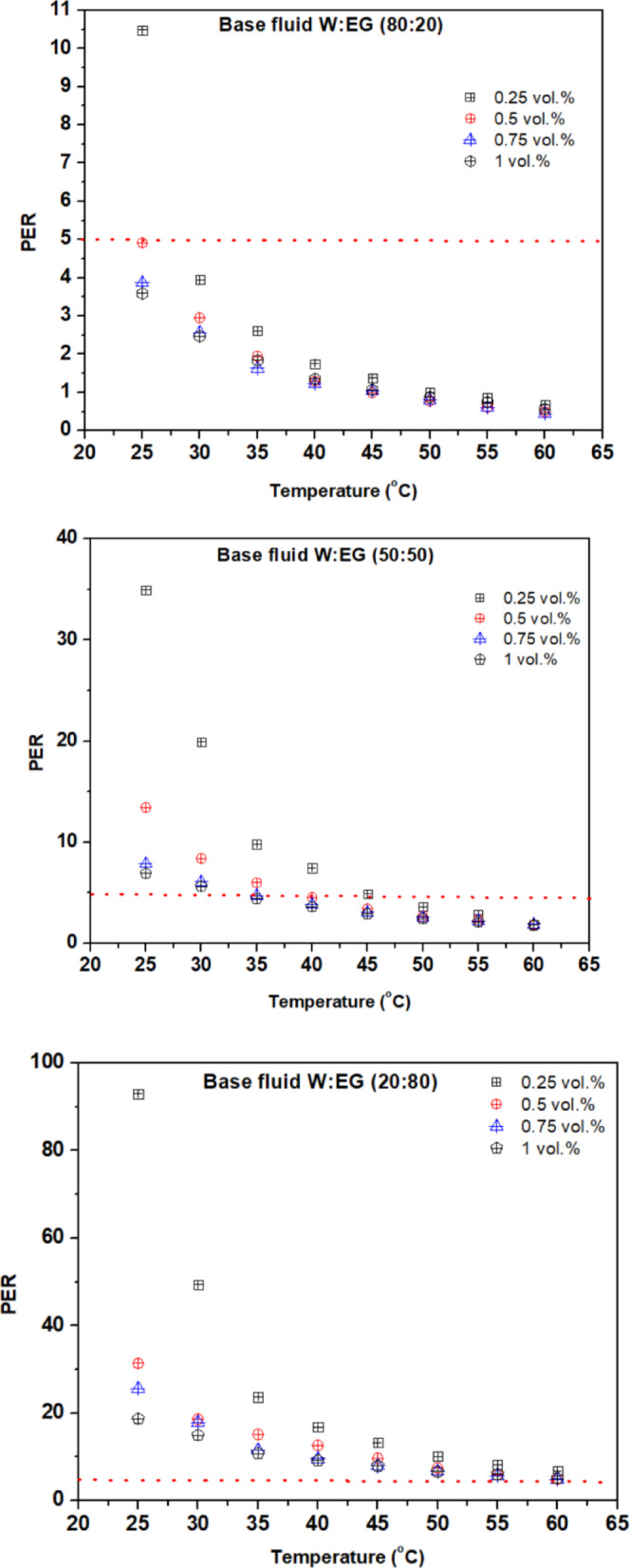
Variation of PER of nanofluids with temperature for (**a**) 80:20, (**b**) 50:50, and (**c**) 20:80 base fluid blend ratio.

PER is a key metric for assessing the heat transfer efficiency of nanofluids, balancing viscosity changes against gains in thermal conductivity and it is estimated using equation provided in the reference^44^. Nanofluids with PER values below 5 exhibit excellent heat transfer performance. Figures 7(a)–(c) highlight PER variations for hybrid nanofluids under different base fluid ratios and temperatures. An increase in EG content leads to higher viscosity and lower thermal conductivity, indicating that water-rich mixtures are preferable at low to moderate temperatures, while EG-dominant fluids perform better at elevated temperatures in PEMFCs. Further experimental studies are necessary to validate these trends and refine nanofluid formulations for optimal thermal management.

## Machine learning based model prediction

### Correlation based data analysis

**Fig. 8:**
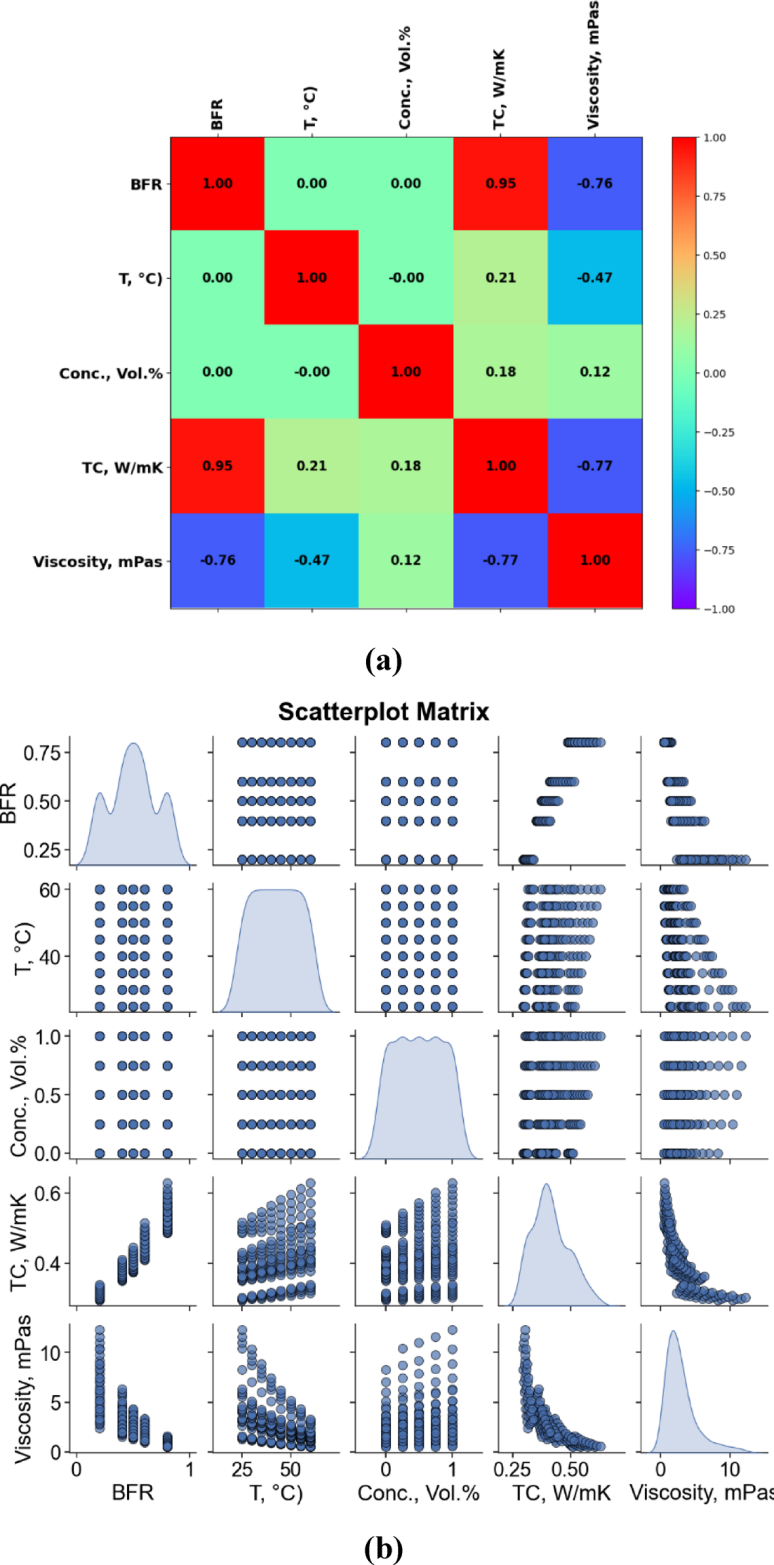
Correlational (**a**) heatmap (**b**) Pair-plot.

The correlation heatmap and scatterplot matrix together provide insight into the relationships between key variables such as Base Fluid Ratio (BFR), Temperature (T), Concentration (Vol.%), Thermal Conductivity (TC), and Viscosity and presented in Fig. 8. From the heatmap(Fig. 8 (a)), it is evident that BFR has a strong positive correlation with thermal conductivity (*r* = 0.95), indicating that increasing the base fluid ratio significantly enhances heat transfer performance. Conversely, BFR shows a strong negative correlation with viscosity (*r* = − 0.76), suggesting that higher BFR reduces fluid resistance, which is desirable for efficient flow. Temperature has a weak positive correlation with thermal conductivity and a moderate negative correlation with viscosity, implying that higher temperatures slightly improve conductivity while reducing viscosity. Concentration appears to have minimal influence on both thermal conductivity and viscosity, as indicated by its weak correlations.

The scatterplot matrix (Fig. 8 (b)) visually confirms these trends. Clear linear patterns are visible between BFR and TC, as well as BFR and viscosity. In contrast, scatterplots involving concentration and temperature show more dispersed data points, reflecting their limited direct impact. The diagonal plots reveal the distribution of each variable, with BFR and TC showing more structured trends compared to the relatively uniform spread of concentration and temperature. Overall, BFR emerges as a dominant factor affecting both thermal conductivity and viscosity, highlighting its importance in optimizing fluid performance for heat transfer applications.

### Model prediction

The dataset employed in this investigation was sourced from a structured Excel sheet consisting of three predictor variables and one target output. We used `pandas` to change the data and `numpy` to do the math on it before utilizing these variables. We shuffled the dataset at random to get rid of any bias in the order, and we used “SimpleImputer” to fill in any missing values. We utilized `matplotlib` to make both regression and error plots for display and assessment. We investigated eight regression models in all. Two of the most important baseline models were LR and DT. We used `sklearn.linear_model` to build LR and optimized `fit_intercept`. We changed the `max_depth` parameter of the DT model, which came from `sklearn.tree`. We used statistical measures including Mean Squared Error (MSE), Coefficient of Determination (R²), and Kling-Gupta Efficiency (KGE) to check how well the model worked. This made sure that the assessment framework was strong. We also utilized the `xgboost` library to deploy the XGBoost model, which included setting the hyperparameters `n_estimators`, `max_depth`, and `learning_rate`. All of the model metrics were stored in a single CSV file, and the prediction and error plots were sent to the local system’s download folder at a high resolution for additional analysis and reporting.

#### Thermal conductivity (TC) model

**Fig. 9:**
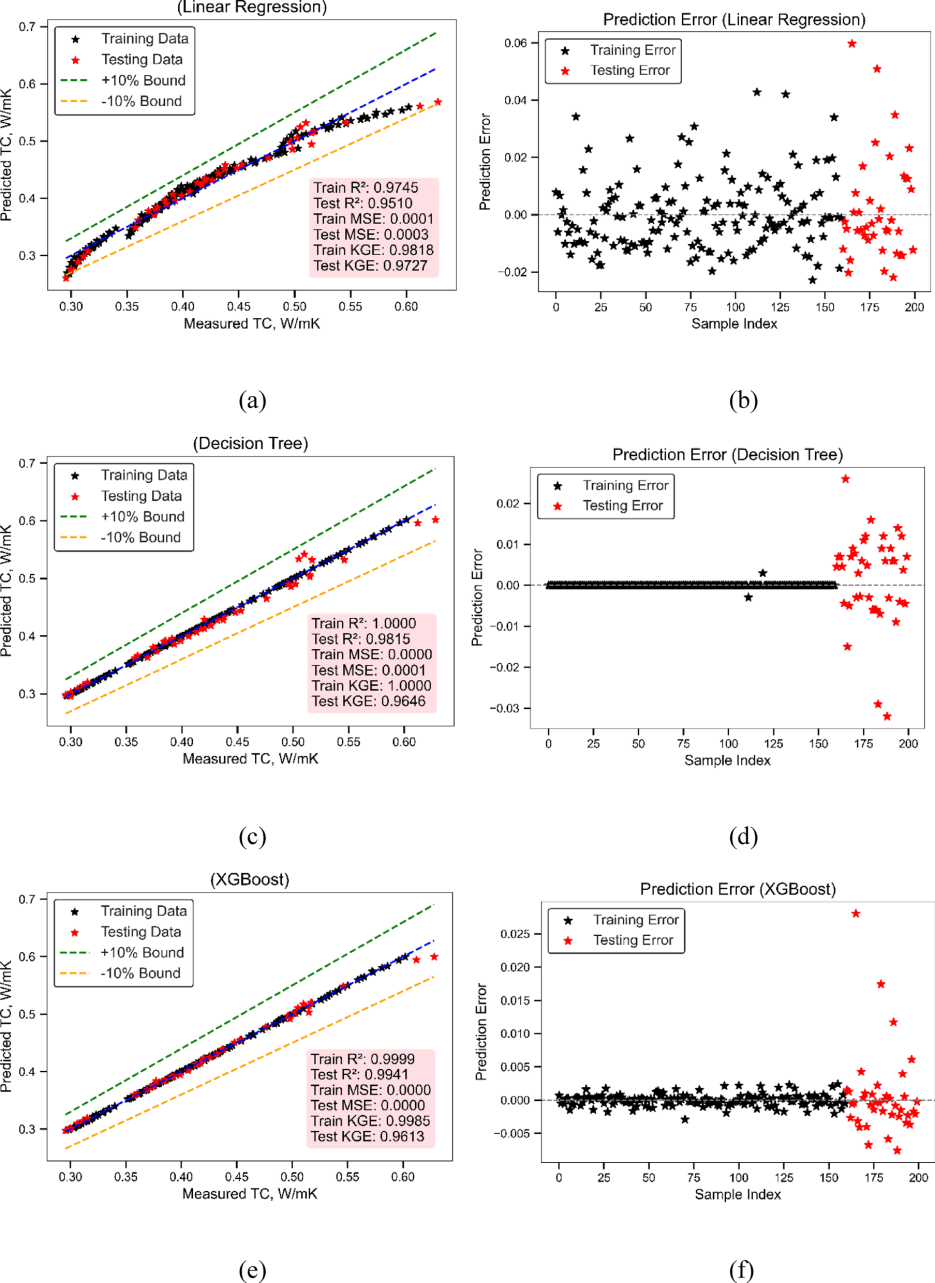
Thermal conductivity model performance (**a**) Actual vs. Predicted values (LR) (**b**) Prediction error (LR) (**c**) Actual vs. Predicted values (DT) (**d**) Prediction error (DT) (**e**) Actual vs. Predicted values (XGBoost) (**f**) Prediction error (XGBoost).

Figures 9(a) and 9 (b) provide insight into the thermal conductivity model’s predictive capability using LR. As evident in Fig. 9(a), the actual vs. predicted scatter plot aligns closely along the 1:1 reference line with the bulk of predictions falling within the ± 10% confidence bounds. The model got a Train R² of 0.9745 and a Test R² of 0.9510, which shows that it worked well in both stages. The MSE values for the Train and Test sets were quite low (0.0001 and 0.0003), which shows that the fit was very good. The Train KGE (0.9818) and Test KGE (0.9727) both show that the model is quite strong and agrees with what was seen. In Fig. 9(b), the residuals for both the train and test sets stay within the range of ± 0.04. They don’t spread out much, and the pattern is mostly unbiased throughout the sample index. This shows that LR was able to accurately capture the linear connection in the thermal conductivity dataset using a simple “fit_intercept = True” setting, without making any major mistakes. The model performance measures in Table 2 show that LR can accurately and generally estimate thermal conductivity. LR is a good foundation model for adding more non-linear features since it stays accurate over folds and sample points, even if it is simple.

Figure 9(c) and 9 (d) indicate that the DT model is better at predicting thermal conductivity values than the other models. Figure 9 (c) shows that both the training and test points are quite close to the 1:1 line, and most of them are within the ± 10% range. DT has very good statistical performance, with a Train R² of 1.0000 and a Test R² of 0.9815. The Train MSE (0.0000) and Test MSE (0.0001) are also very low, which means that the regression splits are quite accurate. The Train and Test KGE values of 1.0000 and 0.9646, respectively, also point to a good correlation, exact magnitude reproduction, and little bias. Figure 9 (d) shows that there is very little residual dispersion in the training predictions (clustered at zero), and the test residuals don’t vary much, keeping generally within ± 0.03. The model’s settings (Table 3) with `max_depth = 10` seem to be just right for capturing the complexity in thermal conductivity data without making it unstable. These signs show how strong and adaptable DT is in mapping correlations in structured datasets of thermal conductivity. There is some difference between test residuals and LR, however, DT always maintains pattern integrity and makes predictions more accurate in thermal models.

Using ensemble boosting, Figs. 9 (e) and 9 (f) show how well the XGBoost model predicts thermal conductivity. Figure 9(e) shows that the actual and projected data points are quite close to each other, with all of them clustered firmly around the 1:1 diagonal. Almost all of the test and train samples are inside the ± 10% range, and the metrics are excellent: Train R² is 0.9999 and Test R² is 0.9941, both of which are quite close to their theoretical maximum. The MSE values (Train: 0.0000, Test: 0.0000) are the lowest of all the models, and the KGE scores (Train: 0.9985, Test: 0.9613) show that the correlation, variance match, and bias reduction are all very consistent. The test residuals in Fig. 9 (f) are mostly close to zero, and there isn’t much spread even at higher indices. The model’s adjusted settings (Table 3), which include `n_estimators = 100`, `learning_rate = 0.1`, and `max_depth = 5`, seem to work quite well for the thermal conductivity job. XGBoost is the best algorithm for predicting thermal conductivity because it strikes the optimum balance between accuracy and generalization. It does better than LR and DT in both numerical metrics and the distribution of residual errors. This makes it even more suitable for modelling high-fidelity thermal properties in nanofluid or composite systems.

**Table 2:** Model evaluation using statistical metrics.

	ML	Train MSE	Test MSE	Train *R*^2^	Test *R*^2^	Train KGE	Test KGE
Thermal conductivity model	LR	0.0001	0.0003	0.9745	0.951	0.9818	0.9727
	DT	0	0.0001	1	0.9815	1	0.9646
	XGBoost	0	0	0.9999	0.9941	0.9985	0.9613
Viscosity model	LR	1.0571	0.6937	0.8047	0.8561	0.8544	0.828
	DT	0	0.2565	1	0.9468	1	0.9245
	XGBoost	0.0008	0.0269	0.9999	0.9944	0.9991	0.9903

**Table 3:** Model training hyperparameters range and best value.

Model	ML	Hyperparameters Range	Selected Values
Thermal conductivity model	LR	{‘fit_intercept’: ‘[True, False]’}	{‘fit_intercept’: True}
	DT	{‘max_depth’: ‘range(3, 20)’}	{‘max_depth’: 10}
	XGBoost	{‘n_estimators’: ‘range(50, 200)’, ‘learning_rate’: ‘[0.01, 0.1, 0.2]’, ‘max_depth’: ‘range(3, 10)’}	{‘n_estimators’: 100, ‘learning_rate’: 0.1, ‘max_depth’: 5}
Viscosity model	LR	{‘fit_intercept’: ‘[True, False]’}	{‘fit_intercept’: True}
	DT	{‘max_depth’: ‘range(3, 20)’}	{‘max_depth’: 10}
	XGBoost	{‘n_estimators’: ‘range(50, 200)’, ‘learning_rate’: ‘[0.01, 0.1, 0.2]’, ‘max_depth’: ‘range(3, 10)’}	{‘n_estimators’: 100, ‘learning_rate’: 0.1, ‘max_depth’: 5}

#### Viscosity models

**Fig. 10:**
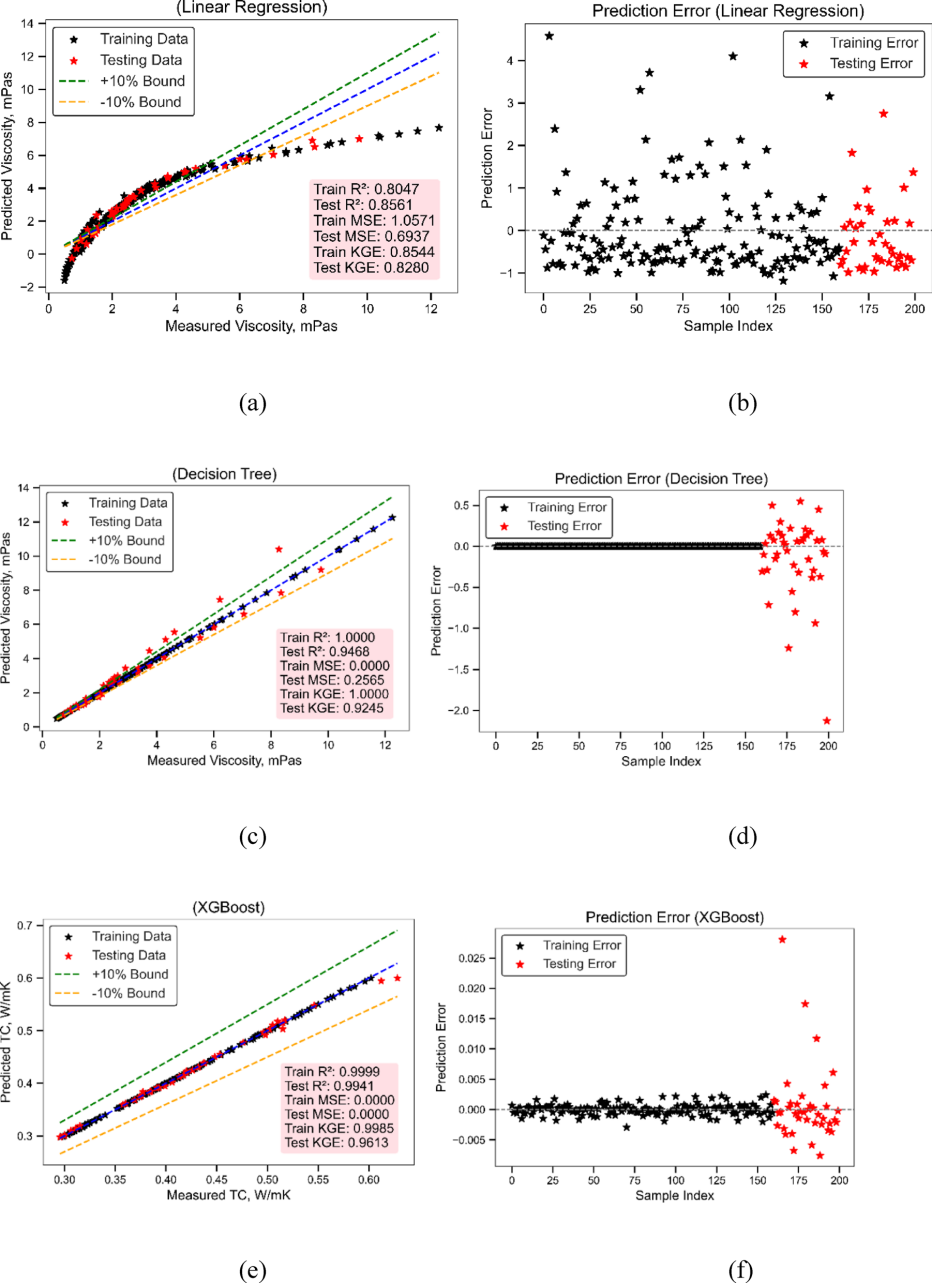
Viscosity model performance (**a**) Actual vs. Predicted values (LR) (**b**) Prediction error (LR) (**c**) Actual vs. Predicted values (DT) (**d**) Prediction error (DT) (**e**) Actual vs. Predicted values (XGBoost) (**f**) Prediction error (XGBoost).

The predictive modeling of viscosity (mPas) using three ML algorithms—LR, DT, and Extreme Gradient Boosting (XGBoost)—is comprehensively illustrated in Figs. 10 (a) to (f). The statistical metrics in Table 2 and the hyperparameter optimization details in Table 3 add to the evidence for these outcomes. The discussion focuses on the model performances by looking at how well they forecast trends, how statistically strong they are, and how consistent they are across training and testing sets. Figure 10 (a) shows the real viscosity values compared to the projected values for the LR model. The LR model only explains things moderately well, with a training coefficient of determination (R²) of 0.8047 and a testing R² of 0.8561. The MSE values for the training and testing sets are 1.0571 and 0.6937, respectively. The KGE values of 0.8544 (train) and 0.8280 (test) further show that the model is only moderately well at capturing correlation, variability, and bias at the same time. Table 3 shows that the LR model used a simple “fit_intercept = True” setting. Some of the test predictions in Fig. 10a are outside the ± 10% range, which shows that adopting a basic linear model to simulate the non-linear behaviour of viscosity has its limits. The error scatter plot in Fig. 10(b) supports this even more. It shows that the residuals are quite spread out, particularly in higher index samples, which suggests that the fit is not good throughout the whole dataset range.

Figure 10(c) and 10(d) illustrate the DT model, which is a big step ahead from LR. It got excellent training accuracy (R² = 1.0000, MSE = 0.0000, KGE = 1.0000) and good generalization on the test set (R² = 0.9468, MSE = 0.2565, KGE = 0.9245). The model works well since its “max_depth” parameter is 10, which was shown to be the best range between 3 and 20 (Table 3). Figure 10 (c) shows that almost all of the predictions are within the ± 10% range, and there is a small group of data points around the optimum fit line. This shows that the method is consistent at capturing nonlinear relationships. The error distribution in Fig. 10 (d) is smaller and more symmetrical than that of LR. This means that there are fewer deviations and that the input-output mapping has been successfully trained. XGBoost, shown in Figs. 10 (e) and (f), had the most accurate predictions of all the models that were tested. As shown in Table 3, the model was set up using `n_estimators` = 100, `learning_rate` = 0.1, and `max_depth` = 5. With this setup, the training R² was 0.9999, the testing R² was 0.9944, the training KGE was 0.9991, and the testing KGE was 0.9903. The MSE values were 0.0008 for training and 0.0269 for testing. These numbers show that XGBoost is the most accurate and balanced algorithm for predicting viscosity. Figure 10(e) shows that the observed and anticipated values are almost exactly the same, with all data points falling within the ± 10% range. Figure 10(f) shows an error profile that is almost flat, with very little change over the sample range. This shows that the predictions are very consistent and have very little bias.

To sum up, LR gives a fundamental baseline that is easy to understand, but it doesn’t show how the data is related in a complicated way. The DT model is better at modelling non-linear data, whereas XGBoost is a little more accurate and stable. Figure 10; Tables 2 and 3 show that XGBoost is the best model for predicting viscosity in this investigation, both statistically and visually.

### Explainable machine leaning based transparent model

**Fig. 11:**
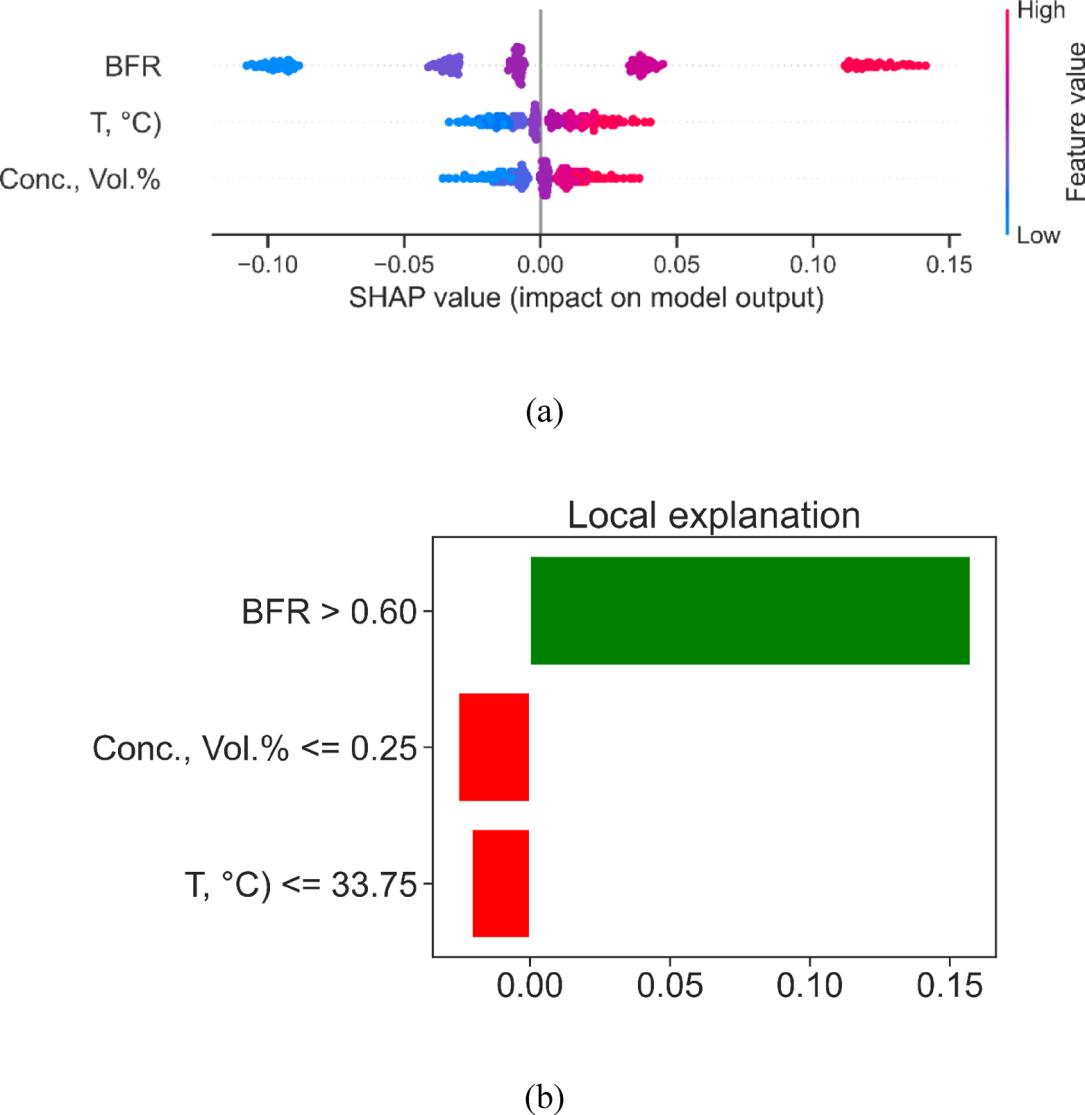
Explainable ML based feature analysis of thermal conductivity model (**a**) SHAP values chart (**b**) LIME based local explanation of feature importance.

Figure 11 illustrates the global and local interpretability of the XGBoost model applied to the prediction task, using SHAP and LIME methodologies, respectively. he top part of Fig. 11a shows a SHAP summary graphic that shows how much each input feature—BFR, T (°C), and Conc. (Vol.%)—adds to the model’s output throughout the whole dataset. The horizontal distance between each point shows the range of SHAP values for each observation, and the colour gradient shows how big the feature value is (blue = low, red = high). BFR is the most important characteristic since greater BFR values are always linked to positive SHAP values, which means that it has a substantial positive effect on model prediction. Next in significance are T (°C) and Conc. (Vol.%), which both display mixed SHAP contributions depending on their value. Lower temperature and concentration levels tend to make the forecast less accurate, whereas higher values either make it more accurate or have less of an effect. The bottom panel (Fig. 11b) shows a LIME-based local explanation for a certain forecast. Here are the three most important rules or choice routes that affect the forecast. The criterion BFR > 0.60 has a big, beneficial effect (green bar), which is in line with SHAP’s general perspective. On the other hand, Conc. (Vol.%) < 0.25 and T (°C) ≤ 33.75 exhibit negative contributions (red bars), which means that these feature values made the anticipated output for this case lower. In general, both interpretability frameworks support the idea that BFR is the main factor in the model’s judgments, with temperature and concentration also playing a role, but only in certain situations. This two-pronged approach makes the model more open and helps optimization tactics that are based on data.

**Fig. 12:**
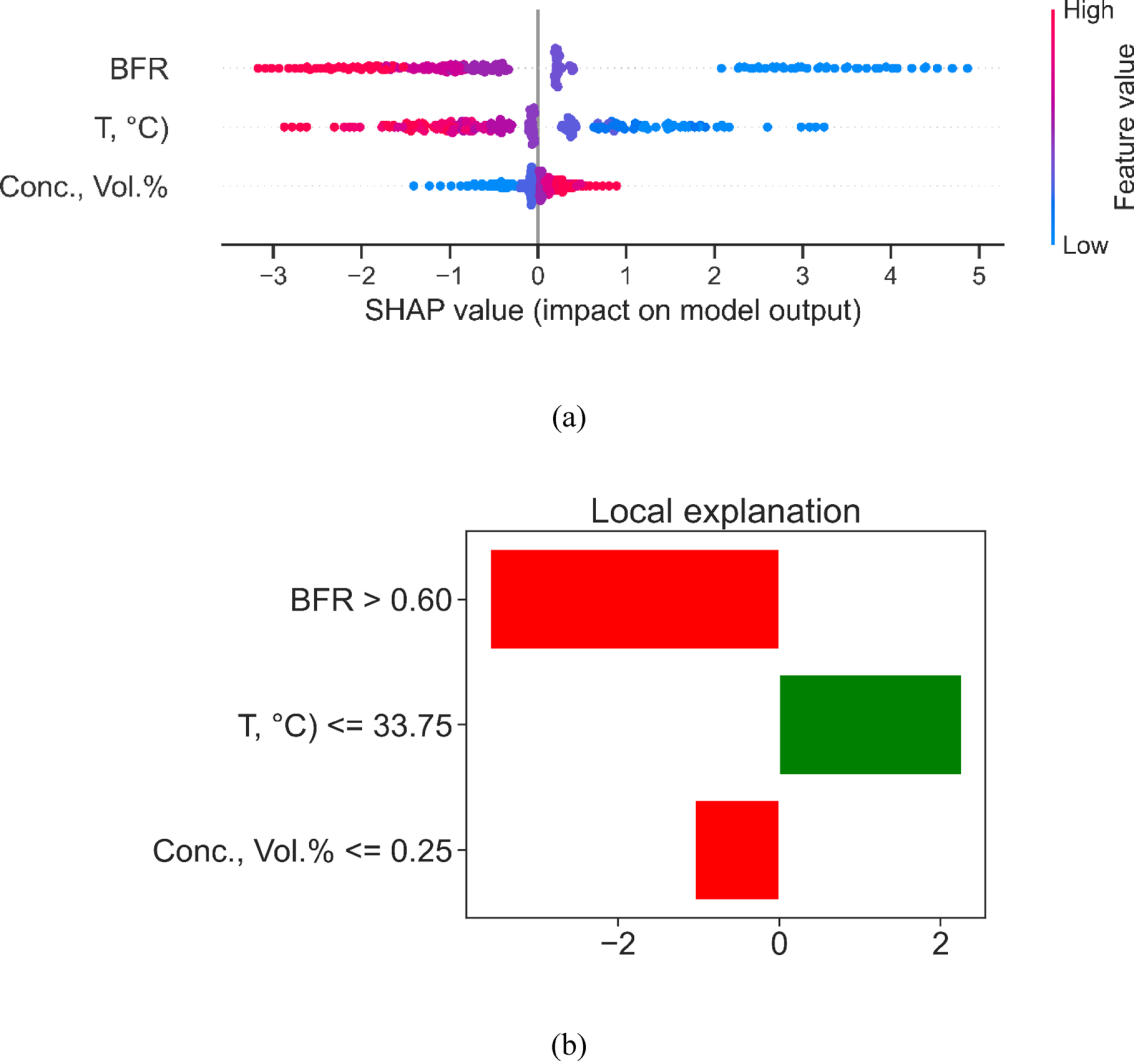
Explainable ML based feature analysis of Viscosity model (**a**) SHAP values chart (**b**) LIME based local explanation of feature importance.

Figure 12 presents a dual-perspective interpretability analysis for the viscosity model, combining global SHAP insights (Fig. 12(a)) with a local LIME breakdown (Fig. 12(b)). In the SHAP summary, each dot represents an individual sample’s contribution to the predicted viscosity, with features ranked by overall influence: BFR exerts the greatest effect, followed by temperature (T, °C) and Concentration (Conc., Vol.%). The horizontal axis quantifies the impact on model output, where values to the right increase predicted viscosity and those to the left decrease it. High BFR (red points) consistently shifts SHAP values toward positive extremes (up to + 5), indicating that elevated filler ratios strongly elevate viscosity. Conversely, low BFR (blue) yields negative contributions (down to − 3), damping viscosity predictions. Temperature exhibits a more symmetrical distribution: high T occasionally raises viscosity (positive SHAP) but often lowers it when paired with other features. Concentration displays subtle but discernible effects, with midrange values clustering near zero impact and extremes causing slight positive or negative shifts.

Beneath the global view, the LIME plot zooms in on one specific instance to reveal feature-level decision rules. Here, the condition BFR > 0.60 appears as a major negative driver (red bar, approximately − 2.5), suggesting that once filler ratio crosses this threshold, the model predicts markedly lower viscosity than baseline. In contrast, T ≤ 33.75 °C contributes positively (green bar, around + 2.0), meaning cooler temperatures in this sample tend to elevate the predicted viscosity. Lastly, Conc., Vol.% ≤ 0.25 exerts a modest negative influence (red bar, about − 1.0), subtly lowering the local prediction. By juxtaposing SHAP’s dataset-wide feature importance with LIME’s case-specific rule contributions, Fig. 12 elucidates both the overarching drivers and the nuanced interactions that govern viscosity predictions, thereby fostering deeper transparency and guiding targeted formulation adjustments.

## Conclusions and future work

The present research focused on optimizing the blend ratio of base fluids (W: EG) for their suitability in PEMFC operations within the temperature range of 25–60 °C.

The study yields several key conclusions:

- An optimum blend ratio of (W: EG) 50:50 and 60:40 was able to produce a homogenous rGO-Al_2_O_3_ hybrid nanofluid with superior dispersion stability (Zeta potential > 44 mV).
- The highest proportion of EG (80%) in base fluid blend produced maximum viscosity ratio about 1.48 at temperatures of 25 °C and concentrations (rGO-Al_2_O_3_) of 1%.
- The temperature rise of hybrid nanofluid (rGO-Al_2_O_3_, 1 vol%) from 25 to 60 °C indicates a strong inverse effect on viscosity with a 71.7% reduction.
- The maximum thermal conductivity ratio of 1.23 at 60 °C for a base fluid ratio of 80:20 with 1 vol% concentration, while the minimum ratio of 1.01 was observed at 25 °C for a 20:80 blend mixture with 0.25 vol% concentration.
- According to the PER analysis, hybrid nanofluid s with a base fluid mixture ratio of 80:20 demonstrate promising results within the considered temperature and concentration range, as indicated by maximum PER values of less than 5.
- The comparative assessment of LR, DT, and XGBoost models for thermal conductivity and viscosity prediction highlighted the superior generalization capability of XGBoost across both cases.
- XGBoost performed better than LR (Test R² = 0.9510) and DT (Test R² = 0.9815) on the thermal conductivity model, with Test R² = 0.9941 and Test KGE = 0.9613. XGBoost once again had the greatest accuracy in the viscosity model, with Test R² = 0.9944 and Test KGE = 0.9903.
- It was significantly better than DT and LR. Both the SHAP and LIME analyses consistently put BFR at the top of the list of most important predictors. In general, combining ML with explainability tools let us model and understand complicated physicochemical interactions with a lot of numerical certainty.

One of the key parameters that contributes to the practical application of rGO-Al_2_O_3_ hybrid nanofluid in PEMFC cooling is the long-term stability of nanofluids with minimal aggregation for a minimum of 1000 h cooling cycle. This is because the stability of hybrid nanofluid directly impacts the cooling efficiency, fuel cell lifespan, and maintenance cost. Therefore, future research should focus on the analysis of nanofluid stability after a minimum of 1000 h cooling cycles. This will provide a breakthrough in understanding the mechanisms governing the correlations between base fluid optimization and thermal properties on the performance of PEMFC. This entails investigating molecular interactions between NPs and base fluids to discern their impact on heat transfer characteristics. Furthermore, integrating computational modelling with experimental studies can provide a comprehensive understanding and aid in predicting optimal formulations for enhanced PEMFC cooling capacity.

## Data Availability

The datasets during and/or analyzed during the current study are available from the corresponding author upon reasonable request.

## Abbreviations

Al₂O₃: Aluminium oxide
BFR: Base fluid ratio
CNT: Carbon nanotubes
CuO: Copper oxide
CTAB: Cetyltrimethylammonium bromide
DT: Decision tree
EG: Ethylene glycol
XGBoost: eXtreme gradient boosting
Fe_2_O_3_: Ferric oxide
Fe₃O₄: Magnetite
GO: Graphene oxide
IEP: Isoelectric point
k: Thermal conductivity (W/m-K)
KGE: Kling-Gupta Efficiency
LIME: Local interpretable model-agnostic explanations
LR: Linear regression
MEA: Membrane electrode assembly
ML: Machine learning
MSE: mean square error
PEMFC: Proton exchange membrane fuel cell
PER: Performance enhancement ratio
R: Base fluid mixture ratio
R^2^: Coefficient of determination
r: Pearson correlation coefficient
rGO: Reduced graphene oxide
SEM: Scanning electron microscopy
SHAP: SHapley additive exPlanations
SiO_2_: Silicon oxide
T: Temperature (^o^C)
T^2^: Square of temperature value
TEM: Transmission electron microscopy
TiO_2_: Titanium oxide
UV: Ultra-violet
V: Volume concentration (%)
Vol.%: Volume concentration
W: Water
XRD: X-ray diffraction

## **Greek Symbols**

$$\mu$$: viscosity (mPa.s)

## **Subscripts**

bf: Base fluid
nf: Nanofluid
